# The Common Systemic and Local Adverse Effects of the Sinovac COVID-19 Vaccine: An Observational Study From Pakistan

**DOI:** 10.7759/cureus.38564

**Published:** 2023-05-04

**Authors:** Hira Khalid Chohan, Aisha Jamal, Muhammad Mubeen, Muhammad Ulusyar Khan, Muhammad Junaid, Musarat Khalid Chohan, Ahmad Imran, Anum Aslam, Adnan Anwar, Atif A Hashmi

**Affiliations:** 1 General Surgery, Dow University of Health Sciences, Karachi, PAK; 2 Internal Medicine, Army Medical College, Rawalpindi, PAK; 3 Internal Medicine, Dow University of Health Sciences, Karachi, PAK; 4 Internal Medicine, Bolan University of Medical and Health Sciences, Quetta, PAK; 5 Anaesthesiology, Jinnah Sindh Medical University, Karachi, PAK; 6 Oncology, Mayo Hospital, Lahore, PAK; 7 Internal Medicine, Liaquat National Hospital and Medical College, Karachi, PAK; 8 Physiology, Hamdard College of Medicine and Dentistry, Karachi, PAK; 9 Pathology, Liaquat National Hospital and Medical College, Karachi, PAK

**Keywords:** burning, swelling, pain, fever, sinovac vaccine

## Abstract

Background

Vaccination acts by boosting the capacity of a person’s immune system to identify and effectively resist infection-causing bacteria and viruses, as it stimulates the immune system to respond to the vaccine’s antigens. The immunological response may include local and systemic symptoms, including pain at the injection site and fever, respectively. The Sinovac vaccine is an inactivated virus vaccine made in China and is one of the most widely used vaccines in many countries; however, the side effects of the Sinovac vaccine have not been well-studied in our population. Therefore, this study assessed the prevalence of side effects experienced by participants after receiving the Sinovac vaccine.

Methodology

This multicenter, cross-sectional study was conducted using a non-probability sampling method. The duration of the study was six months from May 1, 2022, to October 31, 2022. A total of 800 participants who were completely vaccinated with the Sinovac vaccine were included in the study. For categorical data, frequencies and percentages were documented, while for continuous data, such as age, height, weight, and the duration of comorbidities, means and standard deviations were evaluated.

Results

The study findings showed that out of 800 participants, 534 (66.8%) were males and 266 (33.3%) were females, with a mean age of 41.20 ± 13.70 years. Among them, 162 (20.3%) had hypertension, and 104 (13.0%) had diabetes. Following the first dose of the Sinovac vaccine, fever was the most commonly reported side effect in 350 (43.8%) participants. Additionally, pain at the injection site in 238 (29.8%) participants, followed by swelling at the injection site in 228 (28.5%) recipients, were among other common side effects. Following the second dose of the Sinovac vaccine, fever was the most commonly reported side effect in 262 (32.8%) participants.

Conclusions

This study concluded that fever was the most frequent systemic side effect, whereas pain and swelling at the injection site were the most frequent local side effects following the administration of the first and second doses of the Sinovac vaccine. Both dosages of Sinovac were well-tolerated, and the majority of the adverse effects were minor and self-limiting.

## Introduction

The novel coronavirus disease 2019 (COVID-19) was first identified in 2019 in Wuhan, China. The World Health Organization (WHO) confirmed a worldwide crisis over the COVID-19 pandemic on January 30, 2020. This virus rapidly propagated throughout China and has since reached 213 other nations globally. The virus is constantly changing and disseminating by asymptomatic carriers, due to which it was considered a high global health threat. Therefore, the WHO acknowledged on February 24, 2020, that the severe acute respiratory syndrome coronavirus 2 (SARS-CoV-2) can trigger a pandemic and spread globally [1].

Earlier, there were no specific medications and vaccinations for the treatment and prevention of COVID-19. Thus, the only way to control the spread of the virus was to take extensive precautions, including extensive hygiene protocols, regular hand washing, avoiding face-to-face contact, mask-wearing, and isolation from society. Regionally, this pathogen was expanding rapidly. Various nations worldwide banned large crowds of people to control the spread and interrupt the exponential curve [2,3]. Many countries enforced strict isolation to control the devastation of this extremely infectious disease.

The most effective method for halting the spread of infectious illnesses has always been vaccination. For COVID-19, vaccination is considered an appropriate approach to eliminate this epidemic disease speedily [4]. Vaccinations employ various techniques to provide disease protection. The COVID-19 vaccines used four different approaches, namely, viral vectors, RNA, protein subunits, and whole viruses. Different vaccines were used in each of these approaches, such as non-replicating viral vector vaccines such as Oxford-AstraZeneca and Sputnik V; RNA or messenger RNA (mRNA) vaccines such as Pfizer-BioNTech and Moderna; protein subunit vaccines such as Nuvaxovid and Covovax; and whole virus vaccines such as Sinopharm and Sinovac [5].

The Sinovac vaccine is an inactivated virus vaccine made in China and is one of the most widely used vaccines in many countries, even today. The effectiveness of the Sinovac and Pfizer vaccines was 83.5% and 95%, respectively [6,7]. Among all other vaccines, the Sinovac vaccine has a wide coverage rate, and analysis of possible adverse events has been given attention to make it more effective. Based on different clinical trials of the COVID-19 vaccine, the frequently reported side effects were pain and swelling at the injection site, with a rate of recovery within 48 hours post-vaccination, whereas fever or allergic reactions, such as itching and inflammation, were among less severe reactions caused by COVID-19 vaccines [8-10]. Injection site pain was the most common side effect associated with almost all COVID-19 vaccines. There were some other side effects of the Sinovac vaccine, including fatigue, muscle pain, and diarrhea, which were mild and lasted only for two days. Individuals who were given CoronaVac/Sinovac had a lower frequency of fever than other COVID-19 vaccines. The Sinovac vaccine was suggested for individuals 18 years of age and older [10].

Individuals who have been confirmed to have COVID-19 should not be vaccinated until they have recovered from the disease and proper measures have been taken, followed by the end of isolation. The Sinovac-CoronaVac vaccine has two doses (0.5 mL) given intramuscularly [11]. A booster dose of the Sinovac vaccine can be offered to people 60 years of age and older. However, there is no need for an additional booster dose in individuals under 60 years of age. Initially, the aim of the countries was to maximize the two-dose coverage among the population aged 60 years, and thereafter, offer the third additional booster dose, if required, starting with the oldest age groups [12].

Vaccine acceptance and hesitancy were dynamic and fluctuated throughout the pandemic, being most affected by COVID‐19 [13]. The WHO identified vaccine reluctance as one of the top 10 challenges to global health in 2019 even before the pandemic [14]. Hence, this issue was further complicated by the COVID-19 pandemic. It was necessary that individuals get vaccinated, but the worldwide population showed hesitancy toward vaccines. The reason behind vaccine hesitancy was mistrust in research analysis and the vaccines, with regard to the immediate development of vaccines, negative side effects, and other adverse events [15]. Anxiety was also one of the fears that affected public health compliance [16]. Therefore, strategies were devised to improve the acceptance of COVID-19 vaccinations among individuals, which is essential in the prevention of the spread of COVID-19. Causes and factors should be identified that can increase the ratio of vaccine acceptance, especially among workers, who are at high risk of being infected with COVID-19.

Despite the Sinovac vaccine being widely used in some nations, there is a dearth of published evidence to suggest side effects of the Sinovac COVID-19 vaccination. Therefore, this study aimed to investigate the reported adverse effects of the Sinovac vaccine among participants.

## Materials and methods

This multicenter, prospective, cross-sectional study was performed using a non-probability sampling method. The time period of the study was six months from May 1, 2022, to October 31, 2022. Ethical approval was obtained from Essa General Hospital (Essa/24/2023) before conducting the study. A total of 800 participants who were completely vaccinated (first and second doses) with the Sinovac vaccine were included in the study. Participants of either gender aged 18 to 79 years were included in the study. Participants who had previously received another COVID-19 vaccination or who had never received a COVID-19 vaccine were excluded from the study. Moreover, participants who had a fever or active COVID-19 infection were excluded from the study.

Every participant was given a precise explanation of the research objective before being asked to provide their informed consent to begin the questionnaire. For participant data, a structured questionnaire was used. Participants’ gender, age, comorbidities, Sinovac vaccination with both doses, and local and systemic adverse effects following the first and second doses of the vaccine were reported as part of their demographic information. Adverse effects of the vaccine were categorized into systemic and local side effects. Systemic adverse effects included fever, chills, headache, breathlessness, nausea, diarrhea, muscular pain (myalgia), pain in joints, lymphadenopathy, swelling of glands, sore throat, tension, and tiredness. Local side effects included pain, swelling, redness, and burning at the site of injection. Additionally, vital signs and current episodes of respiratory infections were also documented. Furthermore, the degree of participant satisfaction was recorded.

Statistical analysis

The data were analyzed using SPSS Statistics for Windows, Version 26.0 (IBM Corp., Armonk, NY, USA). For categorical data, such as gender, comorbidities, history of COVID-19 infection, and post-vaccination side effects, frequencies and percentages were documented, whereas means and standard deviations were calculated for continuous data, such as age, height, weight, and the duration of comorbidities.

## Results

A total of 800 participants completely vaccinated with the Sinovac vaccine were studied. There were 534 (66.8%) males and 266 (33.3%) females. The mean age of the participants was 41.20 ± 13.70 years. The mean weight and height of the participants were 65.94 ± 14.40 kg and 5.34 ± 0.71 ft, respectively. The mean duration of hypertension and diabetes was 4.91 ± 4.30 years and 4.90 ± 3.82 years, respectively. Out of 800 participants, only 162 (20.3%) had hypertension, and 104 (13.0%) had diabetes. Additionally, only 20 (2.5%) participants had previous exposure to COVID-19, as shown in Table 1.

**Table 1:** Demographic information of participants vaccinated by the Sinovac vaccine (n = 800). SD: standard deviation; COVID-19: coronavirus disease 2019

Variables		Values
Age (years), mean ± SD		41.20 ± 13.70
Weight (kg), mean ± SD		65.94 ± 14.40
Height (ft), mean ± SD		5.34 ± 0.71
Duration of hypertension (years), mean ± SD		4.91 ± 4.30
Duration of diabetes mellitus (years), mean ± SD		4.90 ± 3.82
Gender, n (%)	Male	534 (66.8%)
	Female	266 (33.3%)
Hypertension, n (%)	Yes	162 (20.3%)
	No	638 (79.8%)
Diabetes mellitus, n (%)	Yes	104 (13.0%)
	No	696 (87.0%)
Previously infected with COVID-19, n (%)	Yes	20 (2.5%)
	No	780 (97.5%)

Following the first dose of the Sinovac vaccine, fever was the most commonly reported side effect in 350 (43.8%) participants. Additionally, pain at the injection site in 238 (29.8%) was followed by swelling at the injection site in 228 (28.5%) recipients. Moreover, other side effects were noted such as burning at the injection site in 210 (26.3%), chills in 180 (22.5%), and fatigue in 140 (17.5%) participants. On the other hand, nausea, diarrhea, and lymphadenopathy were the least reported side effects in 70 (8.8%), 70 (8.8%), and 70 (8.8%) participants, respectively, as shown in Table 2.

**Table 2:** The prevalence of side effects after receiving the first dose of the Sinovac vaccine.

Variables	Values	
	Yes	No
Pain at the injection site, n (%)	238 (29.8%)	562 (70.3%)
Swelling at the injection site, n (%)	228 (28.5%)	572 (71.5%)
Redness at the injection site, n (%)	120 (15.0%)	680 (85.0%)
Lymphadenopathy, n (%)	70 (8.8%)	730 (91.3%)
Fever (temperature >37.8 °C), n (%)	350 (43.8%)	450 (56.3%)
Headache, n (%)	104 (13.0%)	696 (87.0%)
Nausea, n (%)	70 (8.8%)	730 (91.3%)
Rashes, n (%)	140 (17.5%)	660 (82.5%)
Burning at the injection site, n (%)	210 (26.3%)	590 (73.8%)
Flu, n (%)	100 (12.5%)	700 (87.5%)
Anxiety, n (%)	110 (13.8%)	690 (86.3%)
Muscle pain (myalgia), n (%)	120 (15.0%)	680 (85.0%)
Fatigue, n (%)	140 (17.5%)	660 (82.5%)
Joint pain, n (%)	130 (16.3%)	670 (83.8%)
Chills, n (%)	180 (22.5%)	620 (77.5%)
Cough n (%)	90 (11.3%)	710 (88.8%)
Swelling of the glands, n (%)	120 (15.0%)	680 (85.0%)
Sore throat, n (%)	130 (16.3%)	670 (83.8%)
Shortness of breath, n (%)	110 (13.8%)	690 (86.3%)
Diarrhea, n (%)	70 (8.8%)	730 (91.3%)
Chest pain, n (%)	90 (11.3%)	710 (88.8%)

Following the second dose of the Sinovac vaccine, fever was the most frequently reported side effect in 262 (32.8%) participants, followed by pain at the injection site (244, 30.5%). Moreover, swelling at the injection site was also observed in 208 (26.0%) participants. Likewise, nausea and cough were the least reported side effects by 20 (2.5%) and 50 (6.3%) participants, respectively, after receiving the second dose, as shown in Table 3.

**Table 3:** The prevalence of side effects after receiving the second dose of the Sinovac vaccine.

Variables	Values	
	Yes	No
Pain at the injection site, n (%)	244 (30.5%)	556 (69.5%)
Swelling at the injection site, n (%)	208 (26.0%)	592 (74.0%)
Redness at the injection site, n (%)	60 (7.5%)	740 (92.5%)
Lymphadenopathy, n (%)	110 (13.8%)	690 (86.3%)
Fever (temperature >37.8 °C), n (%)	262 (32.8%)	538 (67.3%)
Headache, n (%)	110 (13.8%)	690 (86.3%)
Nausea, n (%)	20 (2.5%)	780 (97.5%)
Rashes, n (%)	152 (19.0%)	648 (81.0%)
Burning at the injection site, n (%)	172 (21.5%)	628 (78.5%)
Flu, n (%)	90 (11.3%)	710 (88.8%)
Anxiety, n (%)	80 (10.0%)	720 (90.0%)
Muscle pain (myalgia), n (%)	162 (20.3%)	638 (79.8%)
Fatigue, n (%)	142 (17.8%)	658 (82.3%)
Joint pain, n (%)	140 (17.5%)	660 (82.5%)
Chills, n (%)	152 (19.0%)	648 (81.0%)
Cough, n (%)	50 (6.3%)	750(93.8%)
Swelling of the glands, n (%)	140 (17.5%)	660 (82.5%)
Sore throat, n (%)	80 (10.0%)	720 (90.0%)
Shortness of breath, n (%)	140 (17.5%)	660 (82.5%)
Diarrhea, n (%)	70 (8.8%)	730 (91.3%)
Chest pain, n (%)	100 (12.5%)	700 (87.5%)

**Figure 1:**
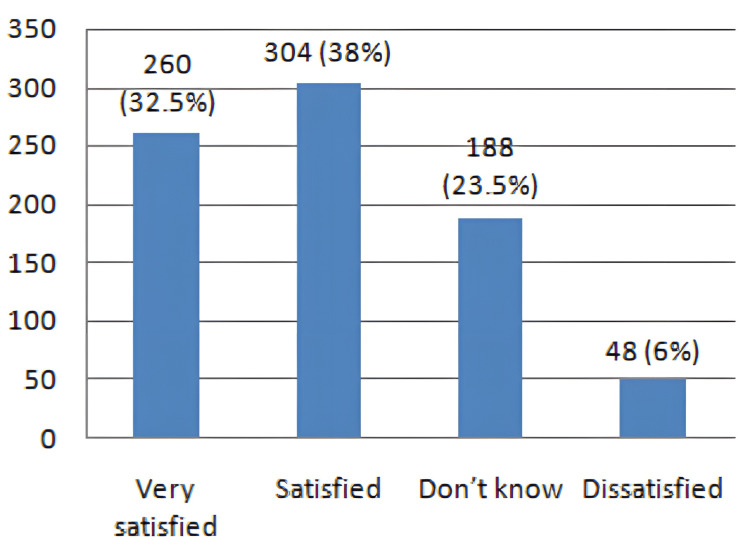
Overall satisfaction level of the participants.

The level of satisfaction with the Sinovac vaccine showed that the majority of the participants (304, 38.0%) were satisfied and 260 (32.5%) were very satisfied with their vaccinations, whereas, 48 (6.0%) participants reported low levels of satisfaction, as shown in Figure 1.

## Discussion

The study was conducted to evaluate the local and systemic side effects of the Sinovac COVID-19 vaccine. We found that the vaccine had overall minor and self-limiting side effects, which were well-tolerated in most individuals. 

Generally, reactions to vaccines in the human body were either negligible or mild. Immunity is induced by vaccination by generating an immune response to the antigens present in the vaccine. The immunological response may include local and systemic symptoms, including pain at the injection site, or raised temperature. The additional constituents of the vaccine, such as adjuvants, preservatives, and stabilizing agents, may provoke reactions. The best immune response is still elicited, even though good vaccines just mildly react [17]. Therefore, this study demonstrated the general and local side effects experienced among Sinovac vaccine recipients.

A national cross-sectional investigation was conducted by Riad et al. among 780 Turkish healthcare professionals who had just received the CoronaVac vaccine. During four weeks post-vaccination, the local and systemic side effects that manifested were noted. Among them, 62.5% of the 780 individuals had a minimum of one side effect. The most frequent local side effects were pain at the injection site (41.5%), whereas the most frequent systemic side effects were tiredness (23.6%), headaches (18.7%), muscular ache (11.2%), and joint pain (5.9%). Local and general side effects notably affected more female medical professionals (67.9%) than their male counterparts (51.4%). A higher risk of CoronaVac side effects was linked to younger age, prior infection, and poor medical conditions (chronic disorders and consistent drug intake) [18]. This study was discordant with our study and indicated that fever was the most frequently reported side effect in 350 (43.8%) participants after receiving the first dose and in 262 (32.8%) participants following the second dose. Moreover, pain and swelling at the injection site were also predominantly reported after both doses. However, fatigue, muscle pain, headache, and joint pain were prevalent at low frequencies. Concerning gender, the vaccinated recipients were predominantly males 534 (66.8%) than females 266 (33.3%).

Similarly, one of the descriptive studies conducted by Batı et al. investigating 355 nurses who received the Sinovac COVID-19 vaccine in Turkey demonstrated that the most frequent regional side effect was pain in 54.6% of the recipients, whereas the most prevalent systemic side effects were fatigue (39.2%) and headaches (34.1%). The majority of those affected were women and adults below the age of 35, with two-thirds of those who received the vaccine reporting at least one local or systemic side effect [19]. Our study did not support the findings of the previously mentioned research, showing that fever, which was common after receiving both doses, was the most common systemic side effect, with more than 30% of participants also reporting pain and swelling at the injection site.

Another observational study was conducted by Nurzak et al. in Indonesia to assess attitudes, perceptions, and practices toward the first Sinovac vaccine in all ages ranging from 18 to 50 years, without experiencing any serious side effects. According to the study, 71 (54.6%) subjects reported that itching and pain were the most frequent side effects of local injections, followed by fatigue in 61 (46%) individuals, muscular pain in (41.5%) subjects, and a negligible number of individuals reported joint pain, nausea, and fever. As many as 60% of patients who reported minor side effects and both local symptoms at the injection site were females. Ages ranging from 18 to 40 years (54.4%) and 41 to 50 years (54.9%) were associated with general symptoms of adverse effects from local injections. Fatigue, joint discomfort, muscle soreness, and nausea were some of the typical side effects, with a vulnerable age range of 41 to 50 years (56.9%). Patients aged 41 to 50 (56.9%) who had general symptoms, such as weariness, joint discomfort, muscular aches, and nausea, were the most common [20]. These findings were not corroborated by our study, which revealed that the mean age of the recipients was 41.20 ± 13.70 years. The most frequently experienced general side effect was fever, and local side effects were pain and swelling at the injection site. Furthermore, chills, fatigue, and muscular pain were noted at a low frequency.

Interestingly, in a cross-sectional analytical investigation in Islamabad by Abbas et al., 205 participants were given 0.5 mL/dosage of the vaccine. Among them, 69 people reported post-vaccination adverse effects, including fever, and 56 of them also had pain, inflammation, and soreness at the injection site. Moreover, 42 participants reported experiencing chills and stiffness, whereas 55 and 28 reported experiencing gastrointestinal distress and symptoms, similar to the flu, respectively. A past history of comorbidities was also closely associated with the occurrence of vaccination side effects. For instance, people with diabetes were more likely than non-diabetic people to experience post-vaccination fatigue (p = 0.049) and symptoms such as flu (p = 0.013) [21]. Our study was concordant with the above-cited study and reported that fever was the most prevalent systemic side effect reported by 350 (43.8%) participants after receiving the first dose and by 262 (32.8%) participants after the second dose. Moreover, pain, swelling, and burning at the injection site were also commonly observed as local side effects. Comorbidities revealed that only 104 (13.0%) recipients were diabetic and 162 (20.3%) had hypertension.

Likewise, our study revealed that fever was the most frequently seen general side effect that was reported in most participants following both doses. Furthermore, the presence of comorbidities, such as hypertension and diabetes, triggered more immune responses causing side effects. Contrarily, Khan et al., in a Pakistani study. included 1,150 recipients who got one of the COVID-19 vaccines, such as Sinopharm, Sinovac, AstraZeneca, and Pfizer (double or booster doses). In their study, burning at the injection site and fever were the most frequent side effects after the first dose reported by 455 (84.3%) and 385 (71.3%) patients with diabetes, respectively. Conversely, in non-diabetics, these symptoms were reported by 252 (41.3%) and 359 (58.9%), respectively, with a significant difference [10].

Similarly, Sinopharm, another comparable immunization that was studied in China by Xia S et al., reported side effects were pain at the injection site (14.3%) and fever (2.4%) instead of fatigue, headache, or muscular pain [22]. In contrast, another research conducted by Khan et al. showed that Pfizer was the most frequently injected vaccine in 187 (34.6%) people with diabetes and Sinovac in 234 (38.4%) non-diabetic people. Regarding side effects, patients with diabetes were more likely to experience swelling, pain, burning at the site of the injection site, and fever than non-diabetes individuals [10]. In our study, fever was the dominant side effect following the Sinovac vaccine, followed by regional swelling and pain. These side effects were mild in intensity and self-limiting.

Interestingly, a cross-sectional analysis by Wang et al. in China investigated the likelihood of SARS-CoV-2 vaccination resistance among people with diabetes. Of the 483 individuals, 273 (56.4%) lacked vaccination belief, including 114 (41.8%) who were doubtful and 159 (158.2%) who were perplexed. The efficacy of the SARS-CoV-2 vaccine and the risk of adverse reactions were the main concerns of individuals who were reluctant to get vaccinated [23]. These results, which were relatively comparable to those of the previously mentioned study by Khan et al., revealed that roughly 345 (63.9%) people with diabetes and 312 (51.1%) people without diabetes were satisfied with their vaccines and exhibited no hesitation to get immunized. On the other hand, 12 (2.2%) diabetic and 14 (2.3%) non-diabetic recipients were discontent due to the presence of adverse reactions [10]. In our study, the level of satisfaction with the Sinovac vaccine showed that the majority of the participants (304, 38.0%) were satisfied and 260 (32.5%) participants were very satisfied with their vaccinations, whereas 48 (6.0%) participants were not satisfied with their vaccinations.

Limitations of the study

Our study had a few limitations. First, the study was unable to relate the effectiveness of the COVID-19 vaccine with the inherent resistance that develops after a COVID-19 infection, as it has been revealed in numerous studies that the natural protection provided by COVID-19 is more defensive, persists for an extended period, and does not have any clotting side effects [24-26]. Second, the existence of side effects may have influenced the willingness of the participants or their ability to respond to the survey. Additionally, the study covered only a few hospitals and vaccination centers in the city and may not be representative of the entire population. Therefore, more large-scale studies on commonly used COVID-19 vaccines should be conducted in this part of the world.

## Conclusions

We found that fever was the most common systemic side effect, whereas pain and swelling at the injection site were the most common local side effects following the administration of the first and second doses of the Sinovac vaccine. Both dosages of Sinovac were well tolerated, and the majority of the adverse effects were self-limiting. These adverse effects can be resolved by resting, eating a healthy diet, and giving patients antipyretics to alleviate their fever and pain. Furthermore, it was shown that the Sinovac vaccine commonly results in minor and self-limiting side effects.
